# Psychophysiological characteristics of pediatric posttraumatic stress disorder during script-driven traumatic imagery

**DOI:** 10.3402/ejpt.v6.25471

**Published:** 2015-02-05

**Authors:** Veronica Kirsch, Frank H. Wilhelm, Lutz Goldbeck

**Affiliations:** 1 Clinic for Child and Adolescence Psychiatry and Psychotherapy, University of Ulm, Ulm, Germany; 2 Department of Psychology, Division of Clinical Psychology, Psychotherapy, and Health Psychology, University of Salzburg, Salzburg, Austria

**Keywords:** Psychophysiology, posttraumatic stress disorder, trauma, electromyography, autonomic nervous system, idiosyncratic trauma script

## Abstract

**Background:**

Psychophysiological alterations such as elevated baseline levels and hyperresponsivity in cardiac, electrodermal, and facial muscle activity have been observed in adults with posttraumatic stress disorder (PTSD). There are only few, inconclusive studies investigating psychophysiological responses in children and adolescents with PTSD.

**Objective:**

This cross-sectional study sought to examine if autonomic variables, facial electromyography (EMG), and self-reported anxiety at baseline, while listening to neutral and idiosyncratic trauma scripts, differ between minors with a trauma history and PTSD, and a traumatized control (TC) group without PTSD. A better understanding of psychophysiological reactions in trauma-exposed children and adolescents could improve differential assessment and treatment decisions.

**Method:**

PTSD was assessed using the Clinician Administered PTSD Scale for Children and Adolescents in 6- to 17-year-old trauma-exposed children, resulting in a group with PTSD according to DSM-IV (*n=*16) and a TC group without PTSD (*n=*18). Facial EMG, (para-)sympathetic measures (heart rate, electrodermal activity, respiratory sinus arrhythmia), and self-reported anxiety were measured during 5-min baseline, 3-min neutral script, and 3-min idiosyncratic trauma script. Baseline, reactivity (trauma minus baseline), and script contrast (trauma minus neutral) were analyzed by multivariate analyses of variance.

**Results:**

Children and adolescents with PTSD reported more anxiety compared to TC for baseline, reactivity, and script contrast (*ps<*0.021, *ds*>0.59), and showed elevated corrugator supercilii muscle activity for script contrast (*p<*0.05, *d=*0.79). No group differences emerged for sympathetic or parasympathetic measures.

**Conclusions:**

Children and adolescents with PTSD experienced elevated anxiety at baseline and elevated anxiety and facial corrugator muscle response to an idiosyncratic trauma narrative. Autonomic hyperreactivity, typical for adult PTSD samples, did not figure prominently.

Posttraumatic stress disorder (PTSD) is a common mental disorder, leading to severe disruptions in psychosocial functioning. It has been much less studied in children and adolescents compared to adults, possibly due to challenges in ethics and recruitment. Alterations in biological systems are said to be associated with PTSD, because of their functional relationship with stress responding and hyperarousal symptoms that form one diagnostic core feature of the disorder (Langeland & Olff, 2008). Meta-analyses reveal a strong relationship between psychophysiological alterations and PTSD in adults (Buckley & Kaloupek, 2001; Pole, 2007); adults with PTSD have a higher heart rate (HR) at rest, compared to controls with a trauma history but without PTSD (traumatized control; TC) or controls without a trauma history (no-trauma controls). Reactivity of HR, electrodermal activity (EDA), and facial electromyography (EMG) of the corrugator supercilii discriminate between adults with PTSD and TC during exposure to idiosyncratic trauma reminders. Respiratory sinus arrhythmia (RSA) was decreased in PTSD during script-driven imagery compared to TC (Sack, Hopper, & Lamprecht, 2004). Psychophysiological alterations in PTSD seem to be reversible by cognitive behavioral therapy, emphasizing their potential as a marker of symptom remission (Blanchard et al., 2002; Lindauer et al., 2006).

Compared to the extensive literature on adult PTSD, investigations of psychophysiological alterations in pediatric posttraumatic stress are rare. The results of the few existing studies are ambiguous, and do not generally replicate findings from adults (Kirsch, Wilhelm, & Goldbeck, 2011). During resting baselines and tasks with non-trauma related stressors no differences between children and adolescents with PTSD and traumatized or no-trauma controls in HR, blood pressure or EDA were observed (Jones-Alexander, Blanchard, & Hickling, 2005; MacMillian et al., 2009; Saltzman, Holden, & Holahan, 2005). During exposure to threat-related pictures, children and adolescents with PTSD showed reduced habituation in skin conductance response compared to no-trauma controls (Grasso & Simons, 2012). The majority of EMG studies on eye-blink responses to sudden bursts of loud noise report exaggerated startle in pediatric PTSD (Grasso & Simons, 2012; Lipschitz et al., 2005; Ornitz & Pynoos, 1989). HR and diastolic blood pressure at rest, during, and after reporting of the traumatic event (mostly motor vehicle accidents) were higher in children and adolescents with a trauma history, regardless of their PTSD status (Saltzman et al., 2005; Scheeringa, Zeanah, Myers, & Putnam, 2004). In blood pressure or HR, no differences between children and adolescents after accidents or no-trauma controls emerged when listening to an audiographed idiosyncratic trauma script (Jones-Alexander et al., 2005). RSA (Scheeringa et al., 2004), a non-invasive measure of cardiac vagal activity, and EDA (Jones-Alexander et al., 2005) were independent of PTSD status during exposure to idiosyncratic trauma-related reminders.

Previous studies with children and adolescents differ with respect to methodological quality in the assessment of psychophysiological variables, which makes it difficult to compare their results. With the exception of HR, only a few studies assessed EDA or RSA, although these variables are reported to be associated with stress, anxiety, and emotion regulation (Wilhelm, Schneider, & Friedman, 2006; Orr & Roth, 2000). Most studies investigated children and adolescents after accidents. They did not include trauma history variables or PTSD resulting from multiple trauma in analyses, even if the trauma type and the characteristics of the traumatic event could be crucial for the development and the severity of psychological symptoms and psychophysiological alterations (Langeland & Olff, 2008). In some studies with adults, differential results were observed: participants with less trauma exposure showed exaggerated autonomic responses, whereas participants with multiple trauma experiences had blunted responses (D'Andrea, Pole, DePierro, Freed, & Wallace, 2013).

A better understanding of psychophysiological alterations in pediatric PTSD would help to improve the diagnostic process, for example, to distinguish between avoidance, dissimulation and absence of stress symptoms, or to identify psychophysiological markers indicative of the need for treatment or useful for treatment prognosis (Blechert, Michael, Grossman, Lajtman, & Wilhelm, 2007; Orr & Roth, 2000). Owing to developmental aspects limiting young children's capacity of discerning and verbalizing internal states (Veneziano, 2009), the assessment of autonomic reactions and facial expression of emotions could aid the differential assessment of PTSD. Psychophysiological markers could help to individually adapt exposure-based interventions of trauma-focused therapy of pediatric PTSD (Cohen, 2010). Bearing in mind age-dependent psychophysiological reactivity (Quigley & Stifter, 2006), and a development-related model of PTSD (Pynoos, Steinberg, & Piacentini, 1999), the generalization of findings in adults to younger samples is not appropriate, highlighting the need for additional studies in children and adolescents.

## Objective

This cross-sectional study, therefore, investigates a variety of autonomic variables, facial EMG, and self-reported anxiety at baseline, while listening to neutral and idiosyncratic trauma scripts. It aims to contribute to a better understanding of psychophysiological reactions in children and adolescents with a history of diverse, mostly multiple traumatic events, who developed PTSD. The study group was compared to children and adolescents with a similar trauma history but without PTSD.

According to former results in PTSD and the diagnostic criteria of DSM-IV, we expect the following patterns in children and adolescents with PTSD compared to controls with a trauma history and no PTSD:

1. More reports of anxiety at baseline, stronger reactivity (defined as difference between trauma script and baseline), and stronger script contrast (defined as difference between trauma and an emotionally neutral script; American Psychiatric Association, 2000).
2. Higher baseline levels, stronger reactivity, and stronger script contrast in HR (Buckley & Kaloupek, 2001; Pole, 2007), SCL (Pole, 2007), non-specific fluctuation (NSF; Grasso & Simons, 2012), and facial EMG (Pole, 2007), as well as lower reactions in RSA (Sack et al., 2004).

## Method

### Participants

**Fig. 1:**
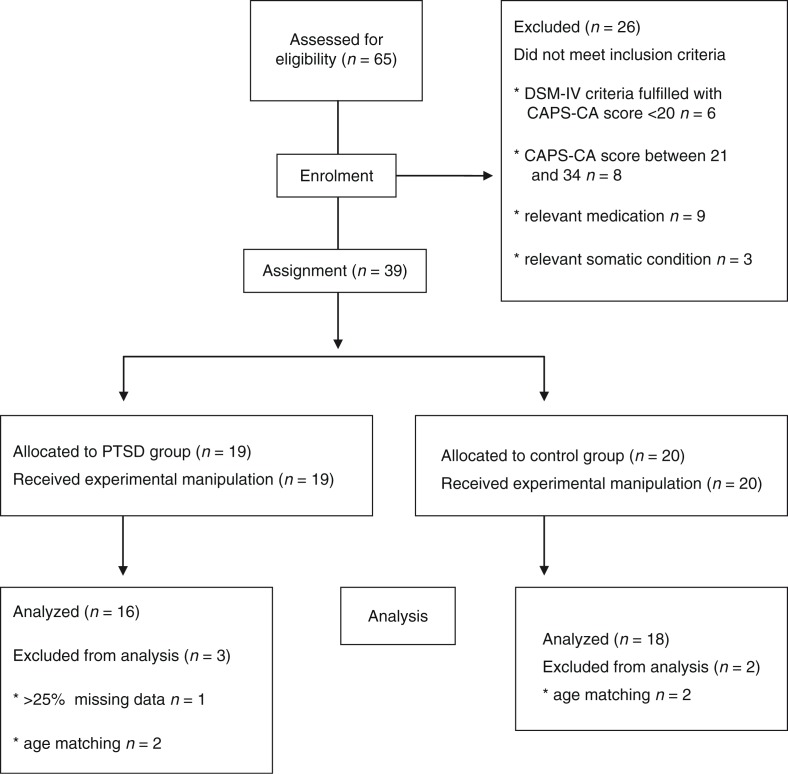
Flowchart of the study.

All participants were consecutively recruited from the Child Trauma Clinic at the Department of Child and Adolescence Psychiatry and Psychotherapy at the University of Ulm, Germany, before getting psychotherapy (if indicated). The following inclusion criteria were applied: a history of one or more traumatic event(s) after the age of 3 years to ensure the child's capacity to verbally remember his traumatic experiences, and dating back at least 3 months to minimize the risk of spontaneous remission (trauma history has been assessed by patients and legal guardians); current age 6–17 years, knowledge of the German language, and current safe living circumstances. Patients were excluded for the following reasons (see Fig. 1): current heart disease, any psychotropic medication, and continuous substance use more than three times a week or within the past 2 days before assessment. To assure contrasting groups regarding the clinical status at the time of assessment, children with a moderate symptom severity score between 20 and 34 points in the Clinician Administered PTSD Scale for Children and Adolescents (CAPS-CA; Nader, Kriegler, & Blake, 2002) were excluded.

The local IRB had approved the study. Informed consent of the parents or legal guardians, and informed assent of children and adolescents were provided in all cases. Participants were reimbursed for their time and travel expenses.

### Procedure

The idiosyncratic trauma scripts were generated based on the CAPS-CA audio-protocol by a clinical psychologist. According to similar investigations in adults (Pitman, Orr, Forgue, deJong, & Claiborn, 1987), scripts were composed as verbatim as possible, in the second-person present tense. They contained context information, physical sensations, emotions, and cognitions the child mentioned during the CAPS-CA. Scripts were read by a person unknown to the minor resulting in approximately 2.5–3.5 min of trauma narrative (see Sample description). The neutral script (3.1 min), which was used for all participants, was drafted according to the characteristics of the trauma script and describes a walk in nature (Pitman et al., 1987). As descriptions of emotions have to be included to match the neutral with the trauma script and emotions could not be described neutrally, words thought to represent mild positive valence like feeling comfortable or content were chosen. A trained investigator conducted the trauma script experiment and psychophysiological assessment on a separate day. The interval between CAPS-CA and psychophysiological assessment did not differ between groups (in days, mean (SD); PTSD: *M=*20.4 (10.28); TC: *M=*17.4 (11.85); *t=*−0.84; *p=*0.40). Prior to the experiment, the investigator explained that the purpose of the study was to find out what happens in their body while listening to stories. Participants were asked to sit down in a semi-reclined armchair in a temperature-controlled room (73°F). The investigator stayed in the room throughout the experiment. Electrodes and sensors were attached and signals were checked. After completing a questionnaire about demographic variables, the following sequence started: 5-min baseline, 3-min neutral script, and about 3-min idiosyncratic trauma script. During each phase, participants were asked to sit comfortably, not to speak or move, and to imagine the stories as vividly as possible. The participants had their eyes closed during all phases. The investigator sat at a 90-degree angle approximately 1.5 m from the child during the psychophysiological recordings. After each phase, the investigator helped the minor to complete a questionnaire about anxiety and other emotions. This lasted a maximum of 2 min during which psychophysiological variables were not recorded.

### Instruments

Posttraumatic stress symptoms were assessed with the CAPS-CA (Nader et al., 2002) by carefully trained assessors. The CAPS-CA has good reliability and validity (German version: interrater reliability 89%; internal consistency Cronbach's *α=*0.91), as well as good construct and content validity (Nader et al., 2002). Picture response options serve as a visual aid to explain the degrees of symptom frequency and intensity. Participants are first asked which traumatic events they had experienced, then to delineate the subjectively most stressful event (index trauma), followed by items assessing frequency and intensity of PTSD symptoms according to DSM-IV on a five-point rating scale. Interviews were audiotaped for the purposes of using the study participant's description of the index trauma and peritraumatic feelings to construct the idiosyncratic trauma scripts.

A child-friendly self-report questionnaire with an 11-point rating scale (0=not at all, 10=extremely; Wilhelm, Schneider, & Friedman, 2006) was completed after each of the three phases. Baseline and difference scores of the item “anxiety” were assessed to monitor self-reported anxiety responses during the experiment. The item “thought about my traumatic event” was assessed to evaluate whether the experimental trauma confrontation worked.

### Psychophysiological outcome variables

The psychophysiological variables assessed cover a range of sympathetically and/or parasympathetically innervated organ systems. HR is dually innervated by sympathetic and parasympathetic branches of the autonomic nervous system and has a track record of being a particularly strong peripheral marker of anxiety (Wilhelm & Roth, 1998). RSA, the rhythmic oscillation of HR with breathing, is driven parasympathetically and has been linked to social affect regulation (Oosterman & Schuengel, 2007; Porges & Furman, 2011). NSF rate of EDA and skin conductance level (SCL), indicators of arousal, are solely sympathetically innervated. In contrast to SCL, NSF is relatively independent of individual structural differences unrelated to sympathetic innervation, like sweat gland density. Facial electromyographic activity (EMG) is deemed to be under mainly reflexive emotional control and is interpreted as a measure of stimulus appraisal and affect-laden information processing (Larsen, Norris, & Cacioppo, 2003; Tassinary & Cacioppo, 1992). The EMG of the corrugator supercilii muscle, in particular, has been shown to vary consistently as a function of negative valence appraisal even if participants try to inhibit their facial response (Tassinary & Cacioppo, 1992). As far as we know, EMG of the corrugator supercilii muscle has not been assessed up to now in children and adolescents with a trauma history during script-driven imagery.

### Psychophysiological measures

The three experimental phases were timed and presented by E-Prime 2.0 Professional (Psychology Software Tools, Sharpsburg, USA) on a notebook PC (using external loud speakers for the neutral and trauma scripts). Timing markers were automatically transmitted to a second notebook PC by a parallel port interface and to a Nexus10 biosignal amplifier (Mind Media, Roermond-Herten, The Netherlands) via optical coupling. Psychophysiological data acquisition was done by the Nexus10 and transmitted to the notebook PC via Bluetooth, allowing for data recording with Dasylab 8.04 (Geitmann Inc., Menden, Germany) at a sample rate of 512 Hz. Data reduction and artifact editing were performed using ANSLAB software (Wilhelm & Peyk, 2005). To control for movement artifacts, an accelerometer summing up vertical and lateral movements was attached to the right shoulder of the participants. Selection of psychophysiological variables, placement of electrodes and sensors, recording, data reduction, and analysis were done in accordance with published guidelines and conventions for psychophysiological research (e.g., Berntson et al., 1997; Fowles et al., 1981). The electrodes were attached in the following order:

#### Electrodermal measures

All participants washed their hands with water. Two Ag/AgCl electrodes (Medcat^®^, Munich, Germany; 0.6 cm diameter) were filled with isotonic electrode gel and attached at the thenar and hypothenar eminences of the non-dominant hand. Children and adolescents were asked not to move their hands or fingers and to place them with the palm up on their thigh or the armrest. The constant voltage difference between the 0.5-volt electrodes allowed for the calculation of the skin conductance level and of increases greater than 0.02 µS from a zero-slope baseline, to calculate non-specific skin conductance fluctuation rate per minute (NSF-rate).

#### Cardiovascular measures

Three disposable Ag/AgCl electrodes (1.5 cm diameter) were attached as standard Lead-II electrocardiogram (ECG). R-waves were automatically identified by ANSLAB, and converted into HR as number of beats per minute. RSA, that is, the rhythmic oscillation of HR with breathing, was quantified as an index of parasympathetic cardiac control by the natural logarithm of the summed Welch power spectral density of Interbeat-intervals between 0.15 and 0.5 Hz, corresponding to the high frequency (HF) range of heart period variability. The frequency cutoffs for the HF band were in line with prior studies in children (e.g., Pine et al., 1998).

#### Electromyographic measures

After cleaning the skin with ethanol, two small disposable electrodes (Tyco healthcare^®^) were attached at the musculus corrugator supercilii of the left eye. The raw signal was highpass-filtered at 28 Hz, rectified, and smoothed with a 16 Hz lowpass filter.

### Data analyses

All psychophysiological data were visually inspected and edited manually if necessary, for example, because of ectopic beats, false-positive R-waves, or movement artifacts. One participant in the PTSD group had to be excluded from the analysis because of 25% missing data due to technical problems (see Fig. 1). For the other interspersed missing data points, the interpolation method described by Stemmler (2012) was used. The percentage of missing psychophysiological data was 1.7%. All statistical analyses were performed with SPSS (IBM SPSS Statistics 22). Significance levels were set at *α<*0.05; all statistical tests were two-tailed. Effect sizes were calculated with Cohen's *d* interpreted as small=0.2, medium=0.5 or large=0.8 (Cohen, 1988).

Due to the character of the study, stratifying by age could not be done from the beginning, and groups differed about 1 year (see Table 1). Most of the assessed psychophysiological variables are age-dependent. Therefore all analyses were done with an age-matched sample. To match age, the age distribution in both groups was visually inspected with help of a stem and leaf plot and four participants (each group *n=*2) were consecutively excluded.

**Table 1:** Sample description of children and adolescents with trauma history and PTSD and a traumatized control group without PTSD (TC)

Variable *M (SD)* or *n* (%)	PTSD (*n=*16)	TC (*n=*18)	Statistic	*p*
Female	11 (68.8)	10 (55.6)	*χ* ^2^ *=*0.624	0.497
Age (years)	12.71 (3.3)	12.67 (2.5)	*t=*−0.045	0.964
In foster care	7 (43.8)	5 (27.8)	*χ* ^2^ *=*0.946	0.475
Parents of foreign origin	6 (37.5)	5 (27.8)	*χ* ^2^ *=*0.366	0.717
Special education school	4 (25.0)	4 (22.2)	*χ* ^2^ *=*0.361	1.000
CAPS-CA total score	55.44 (15.7)	13.44 (5.9)	*t=*−10.083	<0.001***
Reexperiencing	20.94 (5.0)	5.17 (2.9)	*t=*−11.088	<0.001***
Avoidance/numbing	17.00 (7.0)	4.72 (3.6)	*t=*−6.525	<0.001***
Hyperarousal	17.44 (9.1)	3.67 (2.9)	*t=*−5.791	<0.001***
Index trauma *n*	Physical V. 6 Sexual abuse 4 Domestic V. 2 Accident 1 Death 2, other 1	Physical V. 1 Sexual abuse 2 Domestic V. 6 Accident 2 Death 4, other 3	*χ*^2^*=*9.152	0.242
Time since trauma (years)	2.36 (1.4)	1.97 (1.6)	*t=*−0.720	0.477
Interpersonal	13 (81.2)	12 (66.7)	*χ*^2^*=*0.962	0.448
Sequential	9 (56.2)	10 (55.6)	*χ*^2^ *=*0.002	1.000
Victim (instead of witness)	9 (56.2)	5 (27.8)	*χ* ^2^ *=*2.835	0.163
Multiple trauma types	8 (50.0)	5 (27.8)	*χ* ^2^ *=*1.771	0.291

*** Significant at *α<*0.001; CAPS-CA: Clinician Administered PTSD Scale for Children and Adolescents; V.: violence.

Group differences in nominal variables were analyzed with *χ* ^2^ or Fisher's exact test if cells had *n*<5. All metric variables were tested for normal distribution with Kolmogorov–Smirnov tests, and mean differences were analyzed with *t*-tests or Mann–Whitney U-tests as indicated by distribution characteristics.

To examine if children were following instructions during the experiment, group means of all three phases of the item “thought about my traumatic event” were analyzed using the non-parametric Friedman test for repeated measures. Wilcoxon signed-rank tests were used for post-hoc analysis with a Bonferroni correction resulting in a significance level of *p<*0.017.

Two Repeated Measures ANOVAs with each two measurements (baseline to trauma and neutral to trauma script) were performed with the psychophysiological variables to evaluate if participants responded to the experimental trauma confrontation.

We tested the hypotheses of group differences in a dually innervated arousal variable (HR), sympathetic (NSF, SCL), parasympathetic (RSA), and facial EMG activity, and in self-reported anxiety. Three experimental conditions were compared between groups: baseline, reactivity (calculated as difference between trauma and baseline), script contrast (calculated as difference between trauma and neutral script).

To protect against inflation of type I errors, a multivariate analysis of variance was performed for psychophysiological measures, with group, measure, and phase as factors. Assumptions of MANOVA (normal distribution and homogeneity of the covariance matrices) were not fulfilled for self-reported anxiety. Therefore, Kruskal–Wallis ANOVA was used separately for the item for baseline, script contrast, and reactivity.

A Kendall's т correlation was conducted to account for sufficient independence of psychophysiological and self-reported psychological variables at baseline, justifying the inclusion of all variables into analyses adding each relevant information.

## Results

### Sample description

The recruitment resulted in *n=*16 study participants with PTSD according to DSM-IV and 18 individuals in the control group without PTSD. Groups did not differ regarding duration of trauma scripts in minutes (PTSD: *M=*3.02 (0.17), TC: *M=*2.95 (0.27), *U*=133, *p=*0.704). The index trauma types were similar (see Table 1).

### Adherence to script-driven traumatic imagery instruction

There was a statistically significant difference in self-reported thoughts about the traumatic event depending on the experimental phase for children and adolescents with PTSD (*χ* ^2^ (3)=30.94, *p<*0.001) and without PTSD (*χ* ^2^ (3)=40.08, *p<*0.001). Post-hoc analysis showed no significant alterations between baseline and neutral script in PTSD (*Z*=−2.26, *p=*0.024) and TC (*Z*=−1.26, *p=*0.207). From neutral to trauma script thoughts about the traumatic event increased substantially both in children and adolescents with (*Z*=−3.44, *p=*0.001; *M*_neutral_=0.88 (1.89); *M*_trauma_=8.50 (3.46)) and without PTSD (*Z*=−3.65, *p<*0.001; *M*_neutral_=0.39 (1.15); *M*_trauma_=6.44 (3.20)).

### Self-reported anxiety

Children and adolescents with PTSD reported elevated anxiety at baseline and higher script contrast and reactivity compared to TC (see Tables 2 and 3).

**Table 2:** Baseline levels of self-reported anxiety and psychophysiological variables in children and adolescents with trauma history and PTSD and a traumatized control group without PTSD (TC)

Variable	PTSD *n=*16 *M (SD)*	TC *n=*18 *M (SD)*	*F* (1, 36) or *χ* ^2^ (1, 38)	*p*	Δ	Δ 95% CIs	*d*
Anxiety	2.25 (3.17)	0.28 (0.96)	*χ* ^2^ *=*6.292	0.012*	−1.97	(−3.57, −0.38)	0.89
EMG (mV)	3.95 (2.18)	3.64 (1.84)	0.700	0.409	−0.31	(−1.72, 1.09)	0.16
NSF (1/min)	1.39 (1.94)	3.00 (3.46)	2.810	0.104	1.60	(−0.30, 3.51)	0.58
SCL (µS)	3.39 (1.94)	4.09 (2.66)	0.731	0.399	0.69	(−0.96, 2.36)	0.31
RSA (ms^2^)	8.21 (1.16)	8.54 (0.85)	1.790	0.191	0.33	(−0.38, 1.03)	0.34
HR (bpm)	76.62 (8.97)	75.72 (9.79)	0.279	0.601	−0.90	(−7.49, 5.69)	0.10

* Significant at *α*<0.05; Δ=*M* of TC minus *M* of PTSD; CI=confidence interval; EMG=electromyographic activity of the corrugator supercilii muscle; HR=heart rate; NSF=non-specific fluctuation rate of skin conductance; RSA=respiratory sinus arrhythmia; SCL=skin conductance level.

**Table 3:** Reactivity and script contrast of self-reported anxiety and psychophysiological variables in children and adolescents with trauma history and PTSD and a traumatized control group without PTSD (TC)

Variable	Reactivity							Script contrast						
	
	PTSD *n=*16 *M (SD)*	TC *n=*18 *M (SD)*	*F*(1, 36) or *χ* ^2^ (1, 38)	*p*	Δ	Δ 95% CIs	*d*	PTSD *n=*16 *M (SD)*	TC *n=*18 *M (SD)*	*F*(1, 36) or *χ^2^* (1, 38)	*p*	Δ	Δ 95% CIs	*d*
Anxiety	3.75 (4.65)	1.78 (2.90)	*χ* ^2^ *=*5.322	0.021*	−1.97	(−4.65, −0.70)	0.59	4.88 (4.77)	1.72 (2.74)	*χ* ^2^ *=*10.391	0.001***	−3.15	(−5.83, −0.47)	0.85
EMG (mV)	1.76 (3.38)	0.56 (1.86)	1.37	0.251	−1.19	(−3.07, 0.69)	0.46	1.83 (2.98)	−0.01 (1.75)	4.55	0.041*	−1.85	(−3.53, −0.16)	0.79
NSF (1/min)	5.10 (4.59)	2.63 (4.64)	2.63	0.115	−2.47	(−5.70, 0.76)	0.55	3.16 (4.76)	3.02 (3.51)	0.04	0.843	−0.14	(−3.04, 2.76)	0.03
SCL (µS)	1.53 (1.24)	0.95 (0.83)	2.51	0.123	−0.58	(−1.32, 0.17)	0.57	0.88 (0.91)	0.49 (0.61)	2.12	0.155	−0.39	(−0.94, 0.16)	0.53
RSA (ms^2^)	−0.13 (0.79)	−0.01 (0.51)	0.34	0.563	0.13	(−0.33, 0.58)	0.19	0.05 (0.87)	−0.04 (0.54)	0.13	0.718	−0.09	(−0.59, 0.41)	0.13
HR (bpm)	1.60 (6.58)	−0.01 (4.09)	0.81	0.376	−1.59	(−5.38, 2.18)	0.31	2.48 (6.38)	0.44 (3.69)	1.07	0.310	−2.05	(−5.63, 1.55)	0.41

*** Significant at *α=*0.001

* significant at *α=*0.05;

Δ=*M* of TC minus *M* of PTSD; CI=confidence interval; reactivity=difference between trauma script and baseline; script contrast=difference between trauma and neutral script; EMG=electromyographic activity of the corrugator supercilii muscle; HR=heart rate; NSF=non-specific fluctuation rate of skin conductance; RSA=respiratory sinus arrhythmia; SCL=skin conductance level.

### Psychophysiological responding to script-driven traumatic imagery

From baseline to trauma script (reactivity), facial EMG (*F*(1,34)=2.39, *p=*0.018, *d=*0.16), NSF (*F*(1,34)=20.23, *p<*0.001, *d=*0.39), and SCL (*F*(1,34)=43.72, *p<*0.001, *d=*0.58) increased significantly in participants. From neutral to trauma script (script contrast), NSF (*F*(1,34)=17.60, *p<*0.001, *d=*0.36), and SCL (*F*(1,34)=24.78, *p<*0.001, *d=*0.44) were significantly higher (see Table 4).

**Table 4:** Reactivity and script contrast of psychophysiological variables in all participants (*n*=34)

Variable	Reactivity							Script contrast					
	
	Baseline *M (SD)*	Trauma script *M (SD)*	*F*(1, 34)	*p*	Δ	Δ 95% CIs	*d*	Neutral script *M (SD)*	*F*(1, 34)	*p*	Δ	Δ 95% CIs	*d*
EMG (mV)	3.67 (1.89)	4.85 (3.37)	6.24	0.018*	1.19	(2.19, 2.16)	0.16	3.95 (1.93)	4.04	0.053	0.90	(−0.01, 1.81)	0.11
NSF (1/min)	2.23 (2.85)	5.98 (3.73)	20.23	0.001***	3.74	(2.05, 5.44)	0.39	2.97 (3.39)	17.60	0.001***	3.01	(1.55, 4.47)	0.36
SCL (µS)	3.75 (2.33)	4.98 (2.34)	43.72	0.001***	1.23	(0.85, 1.61)	0.58	4.30 (2.29)	24.78	0.001***	0.68	(0.40, 0.96)	0.44
RSA (ms^2^)	8.44 (0.97)	8.38 (0.99)	0.30	0.586	−0.06	(−0.30, 0.17)	0.01	8.38 (0.99)	0.01	0.983	0.01	(−0.25, 0.26)	0.01
HR (bpm)	75.75 (9.13)	76.45 (8.82)	0.58	0.453	0.72	(−1.21, 2.66)	0.02	74.95 (9.51)	2.84	0.102	1.52	(−0.32, 3.36)	0.08

*** Significant at *α=*0.001;

* significant at *α=*0.05;

Δ=*M* of trauma script minus *M* of baseline respectively *M* of trauma script minus *M* of neutral script; CI=confidence interval; reactivity=difference between trauma script and baseline; script contrast=difference between trauma and neutral script; EMG=electromyographic activity of the corrugator supercilii muscle; HR=heart rate; NSF=non-specific fluctuation rate of skin conductance; RSA=respiratory sinus arrhythmia; SCL=skin conductance level.

### Psychophysiological measures

Children and adolescents with PTSD showed significantly higher script contrast of the EMG of the corrugator supercilii muscle than healthy controls (*F*(1, 36)=4.55, *p=*0.041; *d=*0.79). All other group comparisons yielded no significant differences (see Tables 2 and 3 for detailed results).

Some psychophysiological parameters had small to moderate effect sizes with higher responses for the PTSD group, for example, SCL reactivity (*F*(1,36)=2.51, *p=*0.123; *d=*0.57) or script contrast (*F*(1,36)=2.12, *p=*0.155; *d=*0.53), as well as HR reactivity (*F*(1,36)=0.808, *p=*0.376; *d=*0.31) or script contrast (*F*(1,36)=1.07, *p=*0.310; *d=*0.41).

### Association between psychophysiological and self-reported variables

Among all psychophysiological measures, only SCL and NSF were significantly correlated at baseline (т=0.284, *p=*0.020). More thoughts about the traumatic event at baseline were significantly associated with smaller SCL (т=−0.295, *p=*0.029) and NSF (т=−0.387, *p=*0.004; see Table 5). These correlations were considered to be negligible compared to the benefit of additional information, so all variables were included in analyses.

**Table 5:** Correlations between psychophysiological and psychological self-reported variables

Measure	1	2	3	4	5	6	7
Thoughts about traumatic event	–	0.15	−0.03	−0.39**	−0.29*	0.13	−0.15
Anxiety	0.15	–	−0.14	−0.06	0.11	0.01	0.01
EMG (mV)	−0.03	−0.14	–	−0.02	0.18	−0.08	−0.01
NSF (1/min)	−0.39**	−0.06	−0.02	–	0.28*	0.05	0.14
SCL (µS)	−0.29*	0.11	0.18	0.28*	–	−0.09	0.18
RSA (ms^2^)	0.13	0.01	−0.08	0.05	−0.09	–	−0.22
HR (bpm)	−0.15	0.01	−0.01	0.14	0.18	−0.22	–

* Significant at *α*<0.05;

** significant at *α*<0.01;

EMG=electromyographic activity of the corrugator supercilii muscle; HR=heart rate; NSF=non-specific fluctuation rate of skin conductance; RSA=respiratory sinus arrhythmia; SCL=skin conductance level.

## Discussion

This study investigated self-reported anxiety and psychophysiological functioning of children and adolescents with a trauma history during exposure to an idiosyncratic trauma script. We found significant differences between children and adolescents with and without PTSD in self-reported anxiety and in the script contrast of the facial EMG, but not in the autonomic psychophysiological responding during the trauma script experiment. Thus, our expectation that children and adolescents with PTSD would show specific baseline levels or a characteristic psychophysiological reactivity to trauma reminders could not be confirmed, with the exception of facial EMG. During the experiment, study participants with PTSD reported more anxiety already at baseline, that is, without exposure to their trauma script, compared to traumatized children and adolescents without PTSD. As expected, children and adolescents with PTSD then reacted to their trauma script with greater anxiety than their non-PTSD counterparts, evident in both reactivity and script contrast measures.

Concerning psychophysiological variables, we found higher script contrast of the activity of the corrugator supercilii muscle in children and adolescents with PTSD compared to controls without PTSD. The muscle is involved in frowning and is regarded as the principal muscle in the facial expression of unpleasant affects, varying as a function of intensity and sociality of emotional stimuli. It is also interpreted as an indicator of emotional information processing (Wilhelm, Schneider, & Friedman, 2006; Tassinary & Cacioppo, 1992). It corresponds to the diagnostic criterion of emotional burden at exposure to trauma reminders (American Psychiatric Association, 2000) and mirrors the intersection between overt behavior and psychophysiological reactions. Corrugator supercilii EMG has previously been described as specific to adults with PTSD during exposure with idiosyncratic trauma reminders (Pole, 2007). Our study groups did not differ in EMG reactivity to the trauma script referenced to baseline. This indicates that the direct script contrast (trauma script referenced to neutral script) is more sensitive for detecting characteristic facial affective response to the trauma script as it controls for individual differences in facial EMG reactivity when listening to a story and producing mental imagery.

In contrast to results from adults, no psychophysiological group differences were found in HR, RSA, SCL and NSF, although study groups differed considerably both in diagnosis and the amount of PTSD symptoms. HR is considered to be a particularly strong putative peripheral marker for anxiety (Wilhelm & Orr, 1998), but PTSD was not associated with HR in our study group, or in previous studies with pediatric populations. Thus, higher HR at exposure seems to be specific to adults, but not to children or adolescents with PTSD. It is not associated with current symptoms but with individual trauma history (Kirsch, Wilhelm, & Goldbeck, 2011; Buckley & Kaloupek, 2001). RSA is often interpreted as an index of affect regulation capacity therefore suggesting a strong relation with PTSD (Wilhelm, Schneider, & Friedman, 2006). In contrast to this suggestion, and consistent with a study of Scheeringa et al. (2004), our study did not find differences in RSA dependent on PTSD, either at baseline or in response to trauma reminders.

Developmental and clinical differences might contribute to the obvious discordance between marked psychophysiological alterations in adult patients with PTSD and absent or only low psychophysiological reactions in children and adolescents with PTSD. Age-dependent characteristics, such as a higher resting HR and less sympathetic reactivity in children and adolescents compared to adults may cause divergent findings of autonomic reactions (Quigley & Stifter, 2006). Another explanation might be that the severity of psychophysiological alterations may depend on trauma history, for example, amount and diversity of experienced trauma types, amount of symptoms, or duration of PTSD (D'Andrea et al., 2013; Langeland & Olff, 2008). Possibly, psychophysiological alterations show differential patterns according to time since trauma and trauma history. The experimental setting of having the psychologist nearby the children and adolescents during the investigation might be another explanation for low psychophysiological reactions, as studies found that the presence of another human decreased the perceived threat (Coan, Schaefer, & Davidson, 2006).

Although little is known about response coherence between different emotional systems, results of recent studies suggest a certain concordance between physiology and experience, affected by some parameters like age of the study population, amount and valence of elicited emotion (Lench, Flores, & Bench, 2011; Mauss, Levenson, McCarter, Wilhelm, & Gross, 2005). The obvious discordance between increased self-reported anxiety and mostly absent physiological reactivity in our study group may therefore be explained by the mentioned parameters. Another interesting explanation is suggested by findings of subgroups in adult PTSD, reporting subjective distress without any observable psychophysiological responses (Pineles et al., 2013). This is worth of further investigations as there may exist groups with different needs in psychotherapy.

There are some limitations to the interpretation of our results: We powered the study for large effect sizes, as reported in adult studies. Therefore, the sample size was small and statistical power was not sufficient to detect small or moderate effects. Effect sizes suggest that there may be a rather weak or moderate association between psychophysiological parameters and PTSD. The variability of trauma types or time since trauma was large. Moreover, a control group without a history of traumatic events was not included, although in previous trauma script studies, the most significant differences were reported for comparisons of children and adolescents with vs. without a trauma history, regardless of PTSD. Therefore, we cannot answer the question whether just experiencing a traumatic event might explain the variance in psychophysiological reactions, instead of developing PTSD.

## Conclusions

Our finding of a more negative emotional reaction to idiosyncratic trauma script exposure indicated by a higher script contrast of the facial EMG should be further evaluated in longitudinal studies with a larger sample. The assessment of facial EMG is a non-invasive method, which is easy to realize, even during a treatment session (Wilhelm & Grossman, 2010). Future studies should utilize larger samples to allow for subgroup analyses regarding the effects of trauma type, sex or age groups on psychophysiology, as there remain open questions regarding the relationship of psychophysiological alterations and diagnostic criteria in pediatric PTSD.

## Supplementary Material

Psychophysiological characteristics of pediatric posttraumatic stress disorder during script-driven traumatic imagery

Psychophysiological characteristics of pediatric posttraumatic stress disorder during script-driven traumatic imagery
