# Supplementary material for: Psychophysiological characteristics of pediatric posttraumatic stress disorder during script-driven traumatic imagery
Source: Eur J Psychotraumatol. 2015 Feb 5;6:10.3402/ejpt.v6.25471. doi: 10.3402/ejpt.v6.25471 (PMC4320135; doi:10.3402/ejpt.v6.25471)
Supplement: Psychophysiological characteristics of pediatric posttraumatic stress disorder during script-driven traumatic imagery [file EJPT-6-25471-s001.pdf]

Psychophysiologische Merkmale der posttraumatischen Belastungsstörung im Kindes- und Jugendalter bei der Konfrontation mit einem idiosynkratischen Traumaskript

Veronica Kirsch, Frank H. Wilhelm, Lutz Goldbeck

Hintergrund: Psychophysiologische Veränderungen bei posttraumatische Belastungsstörungen (PTBS) sind aufgrund der Nähe zur physiologischen Stressreaktion und der Übererregungssymptome zu erwarten. Bei PTBS im Erwachsenenalter wurden entsprechende psychophysiologische Reaktionen, wie erhöhte Herzrate (HR) in Ruhe, oder stärkere Reaktivität auf idiosynkratische Traumareize in HR, elektrodermaler Aktivität und fazialer Elektromyografie (EMG) des musculus corrugator supercilii wiederholt beschrieben. Zudem erweisen sich diese Veränderungen durch Psychotherapie als reversibel, könnten also eventuell als Marker der Symptomremission genutzt werden. Die Studienlage im Kindes- und Jugendalter ist ungenügend und repliziert diese Befunde nicht eindeutig. Ergebnisse aus der Forschung mit Erwachsenen lassen sich nicht ohne weiteres auf jüngere Altersgruppen übertragen.
Zielsetzung: Genauere Kenntnisse über psychophysiologische Veränderungen bei traumaexponierten Kindern und Jugendlichen könnten den diagnostischen und therapeutischen Prozess erleichtern, z.B. die Differenzierung zwischen Vermeidungsverhalten, Dissimulation und Symptomfreiheit. Diese Studie vergleicht autonome Parameter, faziales EMG und selbstberichtete Angst von Kindern und Jugendlichen, die nach meist multiplen Traumata eine PTBS entwickelten während einer Ruhesequenz, während dem Anhörens einer neutralen Geschichte, sowie eines idiosynkratischen Traumaskripts. Die Kinder und Jugendlichen der Kontrollgruppe (TK) weisen eine ähnliche Traumavorgeschichte auf, entwickelten jedoch keine PTBS.
Methode: Diagnose und Symptomschwere der PTBS wurden bei traumaexponierten Kindern und Jugendlichen zwischen sechs und 17 Jahren mit dem Interview zu Belastungsstörungen bei Kindern und Jugendlichen (IBS-KJ) erhoben und zwei Gruppen gebildet: PTBS (n=16) mit der Diagnose einer PTBS nach DSM-IV und einer Symptomschwere von mindestens 35 Rohwertpunkten im IBS-KJ, sowie die Kontrollgruppe ohne PTBS (TK; n=18) und mit einer Symptomschwere von höchstens 20 Rohwertpunkten im IBS-KJ. Faziale Elektomyografie, (para)sympathische Parameter (Herzrate, Hautleitwiderstand, unspezifische Hautleit-reaktionen, respiratorische Sinusarrythmie) und Angst im Selbstbericht wurden während folgender Phasen erfasst: 5-minütige Ruhesequenz, 3-minütiges Anhören einer neutralen Geschichte, sowie eines 3-minütigen idiosynkratischen Traumaskripts. Mittels ANOVAs für Messwiederholungen (Ruhesequenz zu Traumaskript; neutrales zu Traumaskript) wurde analysiert, ob die Gesamtstichprobe auf die experimentelle traumabezogene Konfrontation reagiert. Um die Unabhängigkeit zwischen physiologischen und selbstberichteten psychologischen Variablen während der Ruhesequenz zu klären wurde eine Kendall's τ Korrelation verwendet. Die Hypothese von Gruppenunterschiede in den erhobenen Parametern wurde mittels einer MANOVA für die Ruhesequenz, Reaktivität (Traumaskript minus Ruhesequenz) und Skript-Kontrast (Traumaskript minus neutrales Skript) berechnet.
Ergebnisse: In der Gesamtstichprobe nahmen von der Ruhesequenz zum Traumaskript das faziale EMG (p<.05, d=.16), unspezifische Hautleitreaktionen (p<.001, d=.39) und der Hautleitwiderstand p<.001, d=.58) signifikant zu. Von neutralem Skript zum Traumaskript erhöhten sich unspezifische Hautleitreaktionen (p<.001, d=.36) und der Hautleitwiderstand (p<.001, d=.44) bedeutsam. Während der Ruhesequenz korrelierten Gedanken an das traumatische Erlebnis und der Hautleitwiderstand bzw. die Anzahl unspezifischer Hautleitreaktionen negativ. Kinder und Jugendliche mit PTBS berichteten im Vergleich zur Kontrollgruppe von mehr Angst während der Ruhesequenz, für Reaktivität und Skript-Kontrast (ps<.021, ds>.59). Sie zeigten stärkere Aktivität im Musculus corrugator supercilii

im Skript-Kontrast (*p*<.05, *d*=.79). In den autonomen Parametern konnten keine Gruppenunterschiede beobachtet werden.
Schlußfolgerungen: Bei Kindern und Jugendlichen mit PTBS konnten mehr Angst in Ruhe, sowie stärkere Angst und erstmals eine größere Aktivität des fazialen Musculus corrugator supercilii als Reaktion auf die Konfrontation mit einem idiosynkratischen Traumaskript beobachtet werden. Autonome Überreaktivität, wie sie bei Erwachsenen zu beobachten ist, war hier kaum nachweisbar. Mögliche Ursachen der Diskordanz zu den Befunden aus dem Erwachsenenalter, sowie zwischen selbstberichteter Angst und physiologischem Ausdruck werden diskutiert.

Schlüsselbegriffe: Psychophysiologie, Posttraumatische Belastungsstörung, Trauma, Elektromyografie, autonomes Nervensystem, idiosynkratisches Traumaskript

Citation: European Journal of Psychotraumatology 2015, 6: 25471 - http://dx.doi.org/10.3402/ejpt.v6.25471
