# Supplementary material for: Psychophysiological characteristics of pediatric posttraumatic stress disorder during script-driven traumatic imagery
Source: Eur J Psychotraumatol. 2015 Feb 5;6:10.3402/ejpt.v6.25471. doi: 10.3402/ejpt.v6.25471 (PMC4320135; doi:10.3402/ejpt.v6.25471)
Supplement: Psychophysiological characteristics of pediatric posttraumatic stress disorder during script-driven traumatic imagery [file EJPT-6-25471-s002.pdf]

Veronica Kirsch, Frank H. Wilhelm, Lutz Goldbeck

Psychofizjologiczne charakterystyki zaburzenia po stresie traumatycznym wśród dzieci i młodzieży uczestniczącej w kontrolowanej ekspozycji na traumatyczne obrazy

Wprowadzenie: Wśród dorosłych osób cierpiących na PTSD obserwuje się wiele różnych zmian natury psychofizjologicznej, takich jak zwiększona reaktywność mięśni sercowych i twarzowych oraz podwyższona aktywność elektrodermalna. Dotychczas niewiele badań poświęcono tego typu psychofizjologicznym zmianom wsród dzieci i młodzieży cierpiącej na PTSD.
Cel: Celem tego badania poprzecznego było sprawdzenie różnic w poziomie zmian określonych zmiennych psychofizjologicznych podczas kontrolowanej ekspozycji na traumatyczne obrazy pomiędzy grupą dzieci i młodzieży cierpiącej na PTSD i grupą dzieci i młodzieży bez PTSD.
Metoda: PTSD mierzone było za pomocą skali CAPS dla dzieci i młodzieży. Porównano wyniki uzyskane w tej skali przez dzieci i młodzież cierpiącą na PTSD (n=16) na tle dzieci i młodzieży bez tego zaburzenia (n=17). Obie grupy zbadano również pod kątem wielu charakterytyk psychofizjologicznych, jak reaktywność mięśni sercowych i twarzowych oraz aktywność elektrodermalna.
Wyniki: Dzieci i młodzież cierpiąca na PTSD wyrózniała się wyższym poziomem lęku oraz wyższą reaktywnością mięśni sercowych i twarzowych w porównaniu do dzieci i młodzieży nie cierpiącej na PTSD. Nie odnotowano natomiast różnic pomiędzy obiema grupami badanych pod względem miar układu sympatetycznego i parasympatetycznego.
Konkluzje: Wśród dzieci i młodzieży cierpiącej na PTSD obserwuje się istotne zmiany w szeregu charakterystykach psychofizjologicznych w porównaniu do dzieci i młodzieży nie cierpiącej na to zaburzenie. W odróżnieniu od osób dorosłych, dzieci i młodzież cierpiąca na PTSD nie wykazuje zmian w układzie sympatycznym i parasympatycznym.

Słowa kluczowe: psychofizjologia, zaburzenie po stresie traumatycznym, trauma, electromiografia, autonomiczny system nerwowy, idiosynkratyczny scenariusz traumy

Name of translator: Marcin Rzeszutek, University of Finance and Management in Warsaw, Poland

Citation: European Journal of Psychotraumatology 2015, 6: 25471 - http://dx.doi.org/10.3402/ejpt.v6.25471
